# Pro-inflammatory pattern of IgG1 Fc glycosylation in multiple sclerosis cerebrospinal fluid

**DOI:** 10.1186/s12974-015-0450-1

**Published:** 2015-12-18

**Authors:** Manfred Wuhrer, Maurice H. J. Selman, Liam A. McDonnell, Tania Kümpfel, Tobias Derfuss, Mohsen Khademi, Tomas Olsson, Reinhard Hohlfeld, Edgar Meinl, Markus Krumbholz

**Affiliations:** Center for Proteomics and Metabolomics, Leiden University Medical Center, Leiden, The Netherlands; Department of Molecular Cell Biology and Immunology, VU University Medical Center, Amsterdam, The Netherlands; Division of BioAnalytical Chemistry, VU University Amsterdam, Amsterdam, The Netherlands; Institute of Clinical Neuroimmunology, Biomedical Center (BMC) and University Hospital, Campus Martinsried-Grosshadern, LMU Munich, Munich, Germany; Departments of Neurology and Biomedicine, University Hospital, Basel, Switzerland; Department of Clinical Neuroscience, Neuroimmunology Unit, Karolinska University Hospital, Stockholm, Sweden; Munich Cluster of Systems Neurology (SyNergy), Munich, Germany; Department of Neurology and Stroke, and Hertie Institute for Clinical Brain Research, University of Tübingen, Tübingen, Germany

**Keywords:** Multiple sclerosis, Cerebrospinal fluid, Immunoglobulin G, Glycosylation

## Abstract

**Background:**

Immunoglobulin G (IgG) effector functions are regulated by the composition of glycans attached to a conserved N-glycosylation site in the Fc part. Intrathecal production of IgG, especially IgG1, is a hallmark of multiple sclerosis (MS), but nothing is known about IgG Fc glycosylation in MS and in cerebrospinal fluid (CSF) in general.

**Methods:**

We applied mass spectrometry of tryptic Fc glycopeptides to analyze IgG Fc glycosylation (sialylation, galactosylation, fucosylation, and bisecting N-acetylglucosamine (GlcNAc)) in 48 paired CSF and serum samples from adult patients with MS or a first demyelinating event highly suggestive of MS (designated as MS cases), and from healthy volunteers and patients with other non-inflammatory diseases (control group). *p* values were adjusted for multiple testing.

**Results:**

Our experiments revealed four main results. First, IgG1 glycosylation patterns were different in CSF vs. serum, in the MS group and even in control donors without intrathecal IgG synthesis. Second, in MS patients vs. controls, IgG1 glycosylation patterns were altered in CSF, but not in serum. Specifically, in CSF from the MS group, bisecting GlcNAc were elevated, and afucosylation and galactosylation were reduced. Elevated bisecting GlcNAc and reduced galactosylation are known to enhance IgG effector functions. Third, hypothesis-free regression analysis revealed that alterations of afucosylation and bisecting GlcNAc in CSF from MS cases peaked 2–3 months after the last relapse. Fourth, CSF IgG1 glycosylation correlated with the degree of intrathecal IgG synthesis and CSF cell count.

**Conclusions:**

The CNS compartment as well as the inflammatory milieu in MS affect IgG1 Fc glycosylation. In MS, the CSF IgG1 glycosylation has features that enhance Fc effector functions.

**Electronic supplementary material:**

The online version of this article (doi:10.1186/s12974-015-0450-1) contains supplementary material, which is available to authorized users.

## Background

Intrathecal immunoglobulin G (IgG) production is a hallmark of multiple sclerosis (MS) [1, 2]. There is strong evidence for IgG-mediated pathomechanisms at least in a subset of patients with MS, although the precise autoantigen remains to be identified for most patients [2–7]. IgG effector mechanisms via complement and Fc gamma receptors (FcγRs) are regulated by the glycan composition at a conserved N-glycosylation site (asparagine 297) in the Fc CH_2_ domain of the heavy chain [8, 9].

**Fig. 1:**
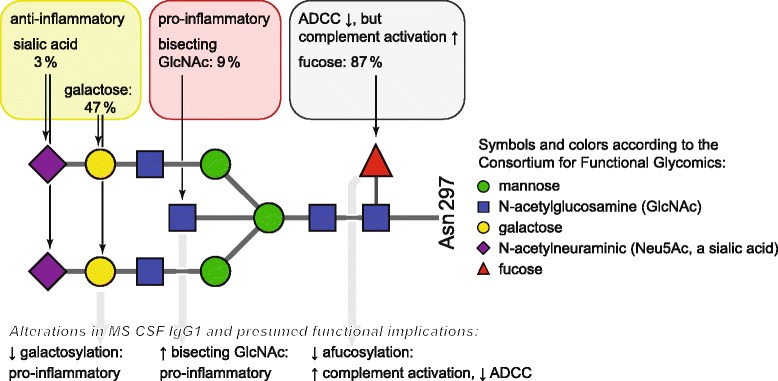
Scheme of the IgG Fc glycan structure and quantitative values for sugar residues in control CSF IgG1. The main variable sugar residues and their presumed pro- and anti-inflammatory properties are indicated: terminal sialic acid (N-acetylneuraminic acid) and galactose residues act mainly anti-inflammatory, whereas bisecting N-acetylglucosamines (GlcNAc) act mainly pro-inflammatory. Core fucosylation decreases ADCC, but in addition was reported to increase complement activation (Gasdaska et al. 2012). Median percentages of the presence of the variable sugar residues on IgG1 in our human control CSF samples are indicated. *Symbols* and *colors* are drawn according to the *Consortium for Functional Glycomics* [55]. *Asn297* asparagine 297, in the Fc part of IgG

The functional in vivo relevance of different IgG Fc glycosylation patterns has been shown in animal models of systemic autoimmune diseases [10–14]. The presence or absence of certain sugar residues (Fig. 1) has been linked to pro- or anti-inflammatory properties: terminal sialylation confers anti-inflammatory properties, and the sialylated fraction of therapeutic intravenous immunoglobulins (IVIG) was suggested to contribute to the therapeutic effect of IVIG [15, 16], although the exact downstream mechanisms may differ between species [12, 17, 18]. Likewise, galactosylation confers anti-inflammatory properties, since decreased galactosylation of IgG resulted in increased pathogenicity in autoantibody-mediated murine models of autoimmune diseases [10, 11, 19]. In contrast, bisecting N-acetylglucosamines (GlcNAc) are pro-inflammatory, e.g., by enhancing antibody-dependent cellular cytotoxicity (ADCC) [8, 20, 21]. Removal of core fucose residues selectively enhances the affinity of IgG for human activating FcγRIIIa, while the binding to all other activating Fc gamma receptors is not affected [8], but complement activation seems to be reduced [22]. Removal of pro-inflammatory glycans by glycosidases such as PNGase F or EndoS abrogates IgG pathogenicity in animal models [13, 14].

Glycosylation of IgG in blood is altered in human autoimmune diseases: in serum from patients with systemic autoimmune diseases such as rheumatoid arthritis, Lambert-Eaton myasthenic syndrome, and Guillain-Barré-Syndrome [23–27], IgG Fc glycosylation is altered toward a more pro-inflammatory pattern. A pro-inflammatory glycosylation pattern precedes clinical disease onset of human rheumatoid arthritis [28]. Importantly, the pathogenic impact of IgG Fc glycosylation has been demonstrated mechanistically with human IgG: anti-aquaporin-4 autoantibodies from patients with neuromyelitis optica (NMO) induce NMO-like lesions in mouse transfer models [29, 30], and this pathogenic effect of NMO-Ig is abrogated by deglycosylation before transfer [31].

The translation into a therapeutic in vivo approach has been pioneered using the IgG-specific endoglycosidase EndoS [32]: in vivo injection of EndoS-diminished MOG_35–55_ induced experimental autoimmune encephalomyelitis as a model of MS [33], as well as anti-GBM and ANCA mediated glomerulonephritis in rodent models [34, 35], and SLE-like disease in BXSB mice [14]. The regulation of IgG effector functions by glycosylation is further utilized by IgG glycol-engineering of therapeutic monoclonal antibodies according to their desired properties [36, 37].

We present the first analysis of IgG glycosylation in the cerebrospinal fluid (CSF) and in MS. We applied mass spectrometry to determine IgG Fc glycosylation in paired CSF/serum samples and addressed the following questions: (1) Does IgG1 glycosylation differ between CSF and serum in the absence of inflammation? (2) Is multiple sclerosis associated with an altered pattern of IgG1 glycosylation in CSF or serum? (3) Is the pattern of CSF IgG1 glycosylation associated with time since last relapse and markers of inflammation in the CSF such as cell count and intrathecal IgG synthesis?

## Methods

### Patients and healthy volunteers

We evaluated 48 paired serum and CSF samples. Thereof, 27 were from patients with definite MS diagnosed according to the 2005 McDonald criteria or a first demyelinating event (“clinically isolated syndrome”, CIS) highly suggestive of incipient MS (MS subgroups, MS-CIS (*n* = 10), relapsing-remitting MS (MS-RR, *n* = 12), secondary progressive MS (MS-SP, *n* = 2), primary progressive MS (MS-PP, *n* = 3); all summarized as MS group). Even though the 2010 McDonald criteria allow for an earlier diagnosis, we adhered to the 2005 criteria since original MRI images were not available for all MS cases for reassessment. Since we were especially interested in CSF IgG, patients were selected for a high intrathecal IgG production according to the Reiber formula [38] (intrathecal fraction of IgG (IF_IgG_) mean 36 %, median 44 %, interquartile range (IQR) 9 to 52 %). From these 27 MS cases, 18 had a recent (<90 days) clinical relapse (median interval 19 days, IQR 8–42 days). Nine patients had received steroids within 90 days, and one patient natalizumab. Two patients had concomitant uveitis, one of whom received methotrexate. We analyzed 21 CSF/serum sample pairs from control donors, including 5 healthy control volunteers (HC) and 16 patients with other, non-inflammatory neurological disease (OND, e.g., tension headache, migraine, pseudotumor cerebri, normal tension hydrocephalus, cerebral ischemia, diabetic neuropathy, and panic disorder). From the donors described, CSF and serum was analyzed, but some mass spectrometric peaks could not be quantified, so that 2 % of data points were missing. From four additional donors (2× MS, 2× OND), serum profiles could be obtained.

Further characteristics of the study cohort are shown in Table 1. Subgroup analysis did not reveal significant differences in glycosylation between the subgroups of the control group (HC and OND) or the MS group (CIS, MS-RR, and MS-CP patients). A change of IgG glycosylation with age has been described in serum [39, 40], and we noted such correlations of IgG glycosylation with age also in CSF IgG. Importantly, however, the similar median age (Table. 1), similar age distribution, and further regression analysis (data not shown) excluded age as a relevant bias for our study.

**Table 1:** Study cohorts: number of samples and clinical characteristics

	MS group	Controls
Number	27	21
Women (percentage)	16 (59 %)	11 (52 %)
Age in years^a^	36 (28–43)	33 (27–48)
Disease duration^a^	1.0 year (36 days–6.9 years)	n.a.
EDSS^a^	2.5 (2, 3)	n.a.
Albumin quotient (*Q*_alb_)^a^	5.4 (4.1–6.8])	4.6 (3.2–7.6)
CSF cell count/μl^a^	13 (3.8–20.2)	2 (1–2.3)

^a^data are given as median (interquartile range)

*EDSS* expanded disability status scale, *n.a.*, not applicable

### Ethics, consent, and permissions

Informed consent was obtained from all patients and healthy volunteers according to local ethics committee regulations (Medical Faculty of the University of Munich, project 159/03; University Erlangen-Nuremburg, project 4203; Karolinska Institute, project Stop MS II, 2009/2107-31/2).

### Basic CSF and serum analysis

CSF cell counts, IgG, and albumin concentrations in CSF and serum were analyzed at each center separately with standard methods as part of the routine patient workup using highly standardized and accurate methods approved for diagnostic use. To ensure accuracy, regular quality controls including round robin tests are performed as applicable. The IgG quotient (*Q*_IgG_) is defined as the ratio of the concentrations of IgG in CSF divided by IgG in serum. As a more elaborate method to quantify the fraction of IgG that is produced intrathecally (IF_IgG_), we applied this formula (${\mathrm{IF}}_{\mathrm{IgG}}=1 - \frac{Q_{\lim}\left(\mathrm{I}\mathrm{g}\mathrm{G}\right)}{Q_{\mathrm{IgG}}}$, with ${Q}_{\lim}\left(\mathrm{I}\mathrm{g}\mathrm{G}\right)=0.93\;\sqrt{{Q_{\mathrm{alb}}}^2+6 \times {10}^{-6}}-1.7\times {10}^{-3}$), which is based on the work of Reiber and Peter [38].

### Mass spectrometry

Serum and CSF were collected in all sites following the same protocol. CSF and serum samples were centrifuged immediately. Aliquots of cell-free CSF and serum supernatant were stored at −80 °C immediately and shipped later on dry ice. Mass spectrometry for the glycosylation profiles of Fc-derived glycopeptides was performed in the same lab and same experimental setup following a recently established protocol [41]. Briefly, IgG was purified from serum or CSF using protein A affinity capturing in the 96-well plate format. Purified IgG was subjected to tryptic digestion, and resulting glycopeptides were desalted by reverse phase-solid phase extraction. Glycopeptides were analyzed using a 9.4 T Apex Q matrix-assisted laser desorption/ionization Fourier transform ion cyclotron resonance mass spectrometer (Bruker Daltonics, Bremen, Germany). An example of the obtained Fc glycosylation profiles of paired serum and CSF samples is shown in Additional file 1: Figure S1. Detected IgG Fc glycopeptide signals were integrated. For both the IgG1 and IgG2 subclass, the sum of signal was set to 100 %. From these data, the abundance of IgG1 and IgG2 Fc N-glycan structural features was calculated, including galactosylation, bisecting N-acetylglucosamine (GlcNAc), sialylation, and core fucosylation. Fucosylation was only assessed for IgG1 and not for IgG2 as several fucosylated IgG2 glycoforms could not be determined due to overlay with IgG4 glycopeptides. Data is presented as percentage for each glycosylation feature: e.g., 47 % galactosylation indicates that 47 % of canonical galactose residues according to the scheme in Fig. 1 were actually present. We always plotted the proportion of the less common form (e.g., sialylation, but afucosylation (~13 %) instead of fucosylation (87 %)).

### Statistics and normalization of IgG glycosylation in CSF and serum

Nonparametric tests were used throughout the manuscript. All tests were two-sided unless indicated otherwise. All statistical tests, adjustments for multiple testing, and plotting of data were performed in R [42].

Comparisons between two groups were calculated by Mann-Whitney *U* test for unpaired samples and by Wilcoxon-signed rank test for paired samples. All *p* values from group comparisons were adjusted for multiple testing (*p*_adj_) across all comparisons (sugar residues and IgG subclasses) as family-wise error rate (Bonferroni correction). When depicting glycosylation as absolute values in CSF and serum separately (=2 compartments), this was also taken into account, resulting in a higher correcting factor for absolute CSF and serum values (4 sugars × 2 IgG subclasses × 2 compartments = 16), compared to CSF/serum ratios (4 sugars × 2 IgG subclasses × only 1 ratio of both compartments = 8). Boxplots were plotted with default whiskers from R (range up to 1.5× IQR below/above first/third quartile).

**Fig. 2:**
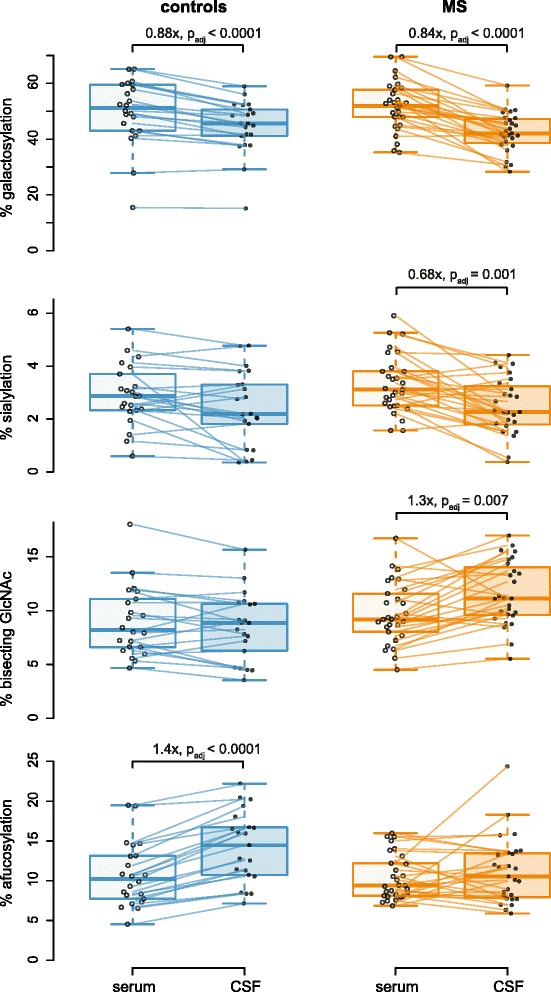
IgG1 glycosylation differs in CSF vs. serum. IgG1 glycosylation was quantified by mass spectrometry in paired CSF/serum samples from 48 donors. Individual data points are shown. *Lines* indicate corresponding CSF/serum pairs, but do not necessarily end directly at the horizontally jittered data points to preserve angles of the connecting lines. Significance was determined using Wilcoxon-signed rank test for paired samples, followed by Bonferroni correction for multiple testing (*p*_ad_j). *Factors* above diagrams indicate fold-changes (medians of paired CSF/serum ratios). Please note that we always show the less abundant glycoform, i.e., afucosylated IgG in the *lowest panel*

Correlations between two parameters were calculated by Spearman’s method. *p* values were adjusted for multiple testing across all possible comparisons of glycoforms and (para-)clinical observations including galactosylation, sialylation, bisecting GlcNAc, afucosylation, age at LP, disease duration, time from last relapse, CSF cell count, *Q*_alb_, *Q*_IgG_, IF_IgG_, EDSS) as false discovery rates [43] separately for each donor group. Consistent with nonparametric correlation statistics, trendlines were computed as robust *locally weighted regression and smoothing scatterplot* (LOWESS) lines [44]. When plotting categorical data on the *x*-axis, data points were jittered horizontally within each category to avoid them obscuring each other. Colors were chosen for best contrast also for colorblind people. We noted that MS cases differed from controls regarding IgG glycosylation in CSF, but not in serum, and normalized CSF glycosylation data as CSF/serum ratio. Using CSF/serum ratios instead of absolute CSF glycosylation values reduced noise (defined as geometric mean of the interquartile ranges of both groups, divided by the difference of medians of both groups) by about 60 %, but did not affect the direction of differences in between the groups (Figs. 2 and 4). Therefore, the CSF/serum ratio for IgG glycosylation was more sensitive and stable, and was used for comparison of groups.

Principal component analysis was performed by the function *princomp* from the *stats* package in R. Principal component analysis axis scales for score and loading were plotted according to the *biplot* standard.

## Results

### CSF vs. serum IgG1 glycosylation differs, both in controls and in MS cases

We quantified IgG1 Fc glycosylation in CSF and serum. A scheme of the attached oligosaccharides, along with frequencies of the respective variable sugar residues in control CSF IgG1, is shown in Fig. 1. A representative mass spectrum is shown in Additional file 1: Figure S1.

We noted differences between CSF and serum IgG1 glycosylation (Fig. 2). In CSF from MS cases, glycans containing bisecting GlcNAc were increased, whereas galactosylated and sialylated species were reduced. Unexpectedly, differences between CSF and serum were detected also in the control group without intrathecal IgG production; in control CSF, galactosylation was reduced as well, while sialylation and bisecting GlcNAc were only slightly (n.s.) shifted into the same direction as in MS CSF. Notably, we observed an increase of afucosylated IgG1 in the CSF of control donors compared to serum that was lacking in the MS cases. Similar results were obtained for IgG2 (Additional file 2: Figure S2).

**Fig. 3:**
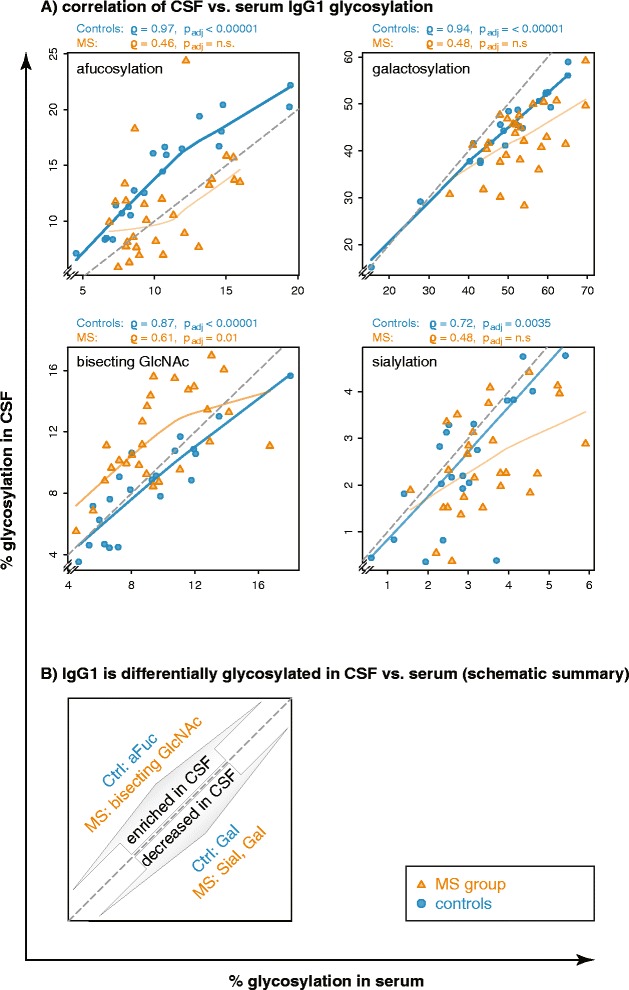
Strong correlation of sugar residues in serum vs. CSF in controls, but much less in the MS group. **a** IgG1 afucosylation, bisecting GlcNAc, galactosylation, and sialylation strongly correlate in CSF vs. serum from control donors (*blue circles*) which do not have intrathecal IgG synthesis. In contrast, the correlation is much weaker within the MS group (*orange triangles*), consistent with additional intrathecal IgG synthesis. *ϱ* and *p* values (Spearman’s method, adjusted for multiple testing) are given for each sugar residue. *Trendlines* represent LOWESS lines (Cleveland 1979) and indicate the strength of association (Spearman’s *ϱ*) by their opacity and thickness. *Dashed gray lines* represent the angle bisectors, indicating where CSF and serum glycosylation would be equal. Thus, each data point that is located *left*/*above* this *line* indicates a sample pair where the respective feature is overrepresented in CSF, whereas each data point *right*/*below* this *line* indicates a sample pair where the respective feature is overrepresented in the serum. This is summarized schematically in **b**, showing that IgG1 is differentially glycosylated in CSF vs. serum. For selection of significant differences between CSF and serum, paired Wilcoxon tests were used with Bonferroni correction for multiple testing (see also Fig. 2). In MS patients (*orange*), bisecting GlcNAc were enriched in CSF, whereas CSF IgG1 was less sialylated and galactosylated compared to serum. In controls (*blue*), CSF IgG1 was also less galactosylated but contained more afucosylated IgG1 (*p*_adj_ < 0.01 for all)

While the *absolute level* of glycosylation differed between CSF and serum, we observed a positive *correlation* between identical IgG1 glycoforms in serum vs. CSF (Fig. 3). This correlation was much higher for the group of control donors (median *ϱ* 0.91, *p*_adj_ < 0.005 for each glycosylation feature) than for the MS group (median *ϱ* 0.48, *p*_adj_ < 0.05 only for bisecting GlcNAc), consistent with an intrathecal IgG production in addition to plasma-derived IgG in the CSF from MS cases. Correlations between serum and CSF IgG2 were less pronounced as compared to IgG1 but present especially for IgG2 galactosylation both in MS and controls (*ϱ* 0.95 and 0.94, *p*_adj_ < 0.00001 for both).

### IgG1 glycosylation is altered in CSF from MS cases vs. controls

**Fig. 4:**
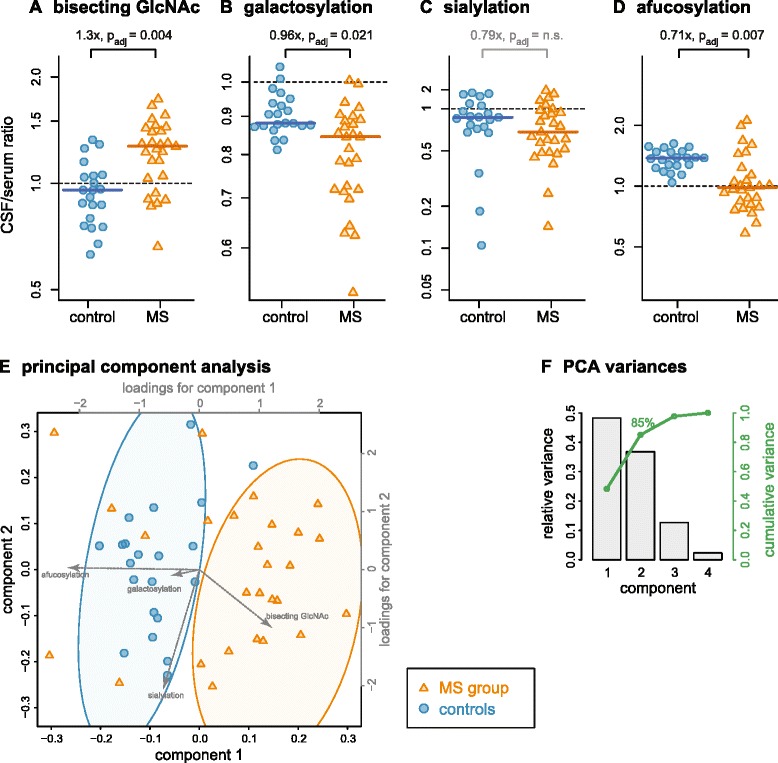
IgG1 Fc glycosylation is significantly altered in the CSF from MS patients vs. controls (elevated; bisecting GlcNAc; reduced; galactosylation and afucosylation). **a-d** Individual glycosylation features. CSF IgG1 glycosylation (normalized to serum IgG1 glycosylation) is displayed. Significance was determined using Mann-Whitney *U* test, followed by Bonferroni correction for multiple testing (*p*_ad_j). *Factors* above diagrams indicate fold-changes. **e** Principal component analysis, incorporating the CSF/serum ratios of all four variable sugar residues, separated the MS from the control group better than each individual sugar residue. *Symbols* represent individual donors (MS; *orange triangles*; controls; *blue circles*). *Dotted gray vectors* represent loadings. **f** Principal component analysis variances. Individual (*bars*, *left axis*) and cumulative (points, *right axis* in *green*) variances for the principal components are shown. In the score plot (**e**, showing the first two components), 85 % of the total cumulative variance is incorporated

Comparing MS cases with controls, IgG1 Fc glycosylation was altered in CSF, but not in serum (Fig. 2). CSF/serum ratios were more sensitive and stable than the absolute CSF values for comparison of groups (see Methods) and are used in the following. *Bisecting GlcNAc* of IgG1 was *increased* in CSF from MS cases. This was evident by the CSF/serum ratio (1.3×, *p* = 0.0005, *p*_adj_ = 0.004; Fig. 4a) and by the absolute CSF values (1.3×, *p* = 0.0021, *p*_adj_ = 0.034; Fig. 2). IgG1 *galactosylation* was slightly but significantly *decreased* in MS CSF (0.96×, *p* = 0.0026, *p*_adj_ = 0.021; Fig. 4b). The decrease of IgG1 *sialylation* (N-acetylneuraminic acid) did not reach statistical significance (Fig. 4c, *p* = 0.08). The CSF/serum ratio of *afucosylated* IgG1 glycoforms was *decreased* in MS compared to control donors (0.71×, *p* = 0.0008, *p*_adj_ = 0.007; Fig. 4d).

Principal component analysis revealed that all four glycosylation features together classified CSF samples better as a MS or control sample than each single glycosylation feature alone, resulting in a good group separation (Fig. 4e). In fact, classification quality according to post-hoc defined criteria (ellipses in Fig. 4e) in our cohort was similar to CSF cell count and intrathecal IgG fraction (principal component analysis; sensitivity 74 %, specificity 100 %; CSF cell count >5 cells; sensitivity 65 % (specificity 100 %, a priori definition of control samples); positive intrathecal IgG fraction; sensitivity 74 % (specificity 100 %, a priori definition of control samples)). Taken together, IgG1 glycofeatures separated our MS patients from controls as good as CSF cell count or intrathecal IgG production, albeit not as sensitive as oligoclonal bands.

**Fig. 5:**
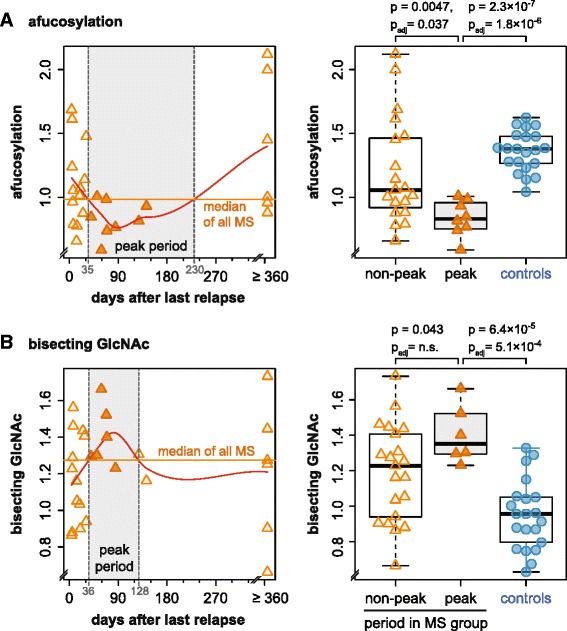
Relationship between glycosylation and time since last relapse. *Left part* (scatterplots); afucosylation (**a**) and bisecting GlcNAc (**b**) in the CSF were plotted against the time since the last relapse. Samples from patients who did not have a relapse within 1 year of sampling were plotted at a cutoff of 1 year. Hypothesis-free regression was computed using the LOWESS method (*red curve*), demonstrating a peak of alterations 2–3 months after the last relapse. The period of the peak, defined by the *crossings* of the *red LOWESS line* with the median of all MS samples (*horizontal line*) was designated as peak period (*gray area*). *Right part* (boxplots): Samples from within this peak period were compared with samples outside of this period, and in addition also with control samples (one-sided *U* test according to the directed hypothesis, followed by Bonferroni correction for the four IgG1 glycofeatures tested)

Next, we analyzed if MS-associated changes of IgG1 glycosylation were more pronounced in a time period related to a relapse. We sought for a hypothesis-free definition for such a period and computed LOWESS regression lines (red curve in Fig. 5). The time between the crosses of their peak with the median of all samples (horizontal line) was designated as the peak period for glycosylation changes. Alterations in afucosylation and bisecting GlcNAc culminated 2–3 months after the last relapse (Fig. 5, left panel) and were significantly more pronounced in samples from within this peak period, compared with samples from outside this period or with control samples (Fig. 5, right panel). Since this analysis was an unplanned subgroup analysis, we did not plan correction for multiple testing, but Bonferroni correction for all four glycosylation features would have left significant results for afucosylation (peak vs. both non-peak and controls) and bisecting GlcNAc (peak vs. controls). In contrast, we could not detect a substantial influence of the time since last relapse on galactosylation and sialylation. Neither could we detect an effect of therapy including steroids on glycosylation, but this study was not powered to detect such effects.

The pattern of alterations between MS CSF and control CSF described here was different from that between control CSF and control serum described in Fig. 3 (summarized in Table 2). In contrast to the differences observed for IgG1 glycosylation in CSF from MS cases vs. controls, such differences for IgG2 glycosylation were present only as a trend or not at all (Additional file 3: Figure S3).

**Table 2:** Summary of alterations in IgG1 glycosylation related to the compartment (CSF vs. serum, see also Fig. 3) and the disease (MS vs. control CSF, Fig. 4a–d, g)

	Control CSF vs. control serum	MS CSF vs. control CSF	Peak after relapse
Afucosylation	↑	↓	y
Bisecting GlcNAc	n.s.	↑	y
Galactosylation	↓	↓	n

*y* yes, *n* no, *n.s.* not significant

### Correlation of IgG glycosylation with CSF cell count and intrathecal IgG fraction

**Fig. 6:**
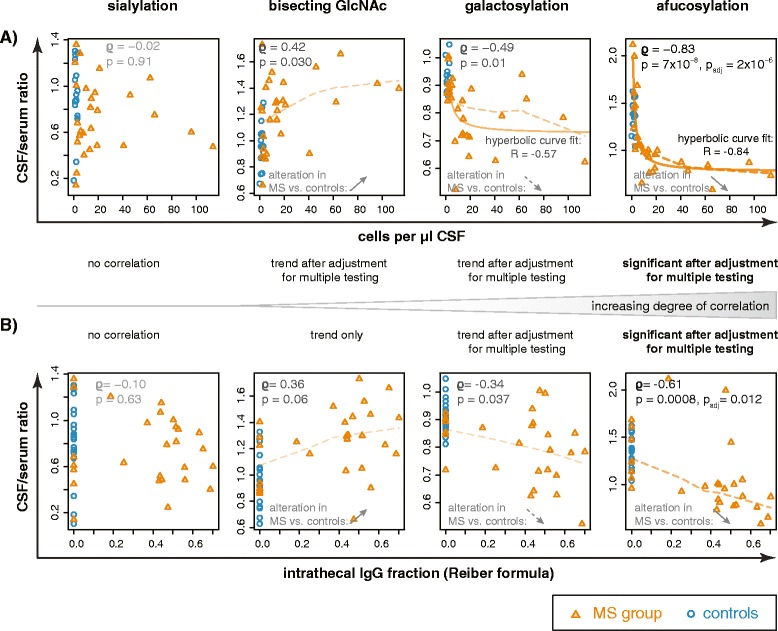
CSF IgG1 glycosylation correlates with intrathecal signs of inflammation within the MS group. IgG1 glycosylation in CSF, normalized to serum, is plotted against the CSF cell count (**a**) and intrathecal IgG fraction calculated by the Reiber formula (**b**) from MS patients (*orange triangles*). For comparison, also data from control donors (*blue circles*) are plotted. Correlation analysis was performed only within the MS group; *ϱ* and *p* values (Spearman’s method) are indicated. *Trendlines* represent LOWESS lines (Cleveland 1979) and indicate the strength of association (Spearman’s *ϱ*) by their opacity and thickness. Where appropriate (galactosylation and afucosylation vs. CSF cell count), an additional hyperbolic model was fitted as indicated. **a** CSF cell count. The strongest correlation was observed for afucosylated IgG1 vs. CSF cell count, which remained significant after correction for multiple testing. The correlation for galactosylation and bisecting GlcNAc was only present as a trend (significant only without adjustment for multiple testing). **b** Intrathecal IgG fraction. Likewise, the strongest correlation was observed for afucosylated IgG1 vs. IF_IgG_, which remained significant after correction for multiple testing. Correlations for bisecting GlcNAc and galactosylation were present only as a trend. **a**, **b** The direction of the correlations paralleled the alteration in CSF from the MS group compared to controls (indicated in the *lower left corner* of each diagram): e.g., a lower fraction of CSF IgG1 was afucosylated in the MS group compared to controls, and this decrease was more pronounced in those patients with a higher-intrathecal IgG synthesis

MS-related changes in Fc glycosylation were more pronounced in those patients with higher cell counts (Fig. 6a). The correlation with CSF cell counts was strongest for afucosylated IgG1 (*ϱ* −0.83, *p* < 0.00001, *p*_adj_ < 0.00001) and present as a trend after correction for multiple testing for galactosylation and bisecting GlcNAc.

We further noted a negative correlation of afucosylated IgG1 with both *Q*_IgG_ and the intrathecally produced IgG fraction (IF_IgG_) according to the Reiber formula [38] (Fig. 6b; *Q*_IgG_; *ϱ* = −0.65, *p* < 0.001, *p*_adj_ = 0.004; IF_IgG_; *ϱ* = −0.61, *p* < 0.001, *p*_adj_ = 0.012). This is consistent with an intrathecal production of less afucosylated IgG, as might be inferred also from the group comparisons (Fig. 4). For IgG1 galactosylation and bisecting GlcNAc, similar trends for an association with *Q*_IgG_ and IF_IgG_ were present and paralleled the observed group differences. Since there is no intrathecal IgG production in control donors, this was only analyzed within the MS group.

There was no significant correlation of any glycosylation feature with disease duration or EDSS, but the study was not powered to detect associations with these clinical parameters.

### Associations between different glycoforms

When assessing different IgG glycosylation features within the *same* compartment (CSF or serum) and *same* group (MS cases or controls), we noted that by far the strongest positive correlation existed between sialylation and galactosylation (for control and MS donors, for CSF and serum IgG1; *ϱ* = 0.67 to 0.93, *p*_adj_ < 0.01 for each; Additional file 4: Figure S4A). In contrast, there was no association for all other combinations of glycoforms (Additional file 4: Figure S4B, exemplified for galactosylation vs. bisecting GlcNAc and afucosylation).

### IgG2 vs. IgG1 glycosylation

The glycosylation pattern was significantly different for IgG2 vs. IgG1 in both serum and CSF from controls and MS cases (reduced; bisecting GlcNAc and galactosylation; elevated in serum; sialylation; Additional file 5: Table S1).

Despite these absolute differences, IgG1 and IgG2 glycosylation was related. In *serum*, the proportions of bisected, galactosylated or sialylated IgG2 correlated with the proportion of the respective IgG1 glycoforms (MS serum; *ϱ* = 0.73 to 0.89, *p* < 0.00005, *p*_adj_ < 0.0005 for all glycosylation features; control serum; *ϱ* = 0.61 to 0.93, *p* < 0.005, *p*_adj_ < 0.05 for all glycofeatures).

In *CSF*, this correlation of respective IgG2 and IgG1 glycoforms was much weaker and only present as a trend for most glycosylation features, especially in the MS group (*ϱ* 0.45 to 0.84 for all; *p*_adj_ < 0.05 for galactosylation and sialylation in control CSF; only as a trend for bisecting GlcNAc in control CSF and all glycosylation features in MS CSF).

## Discussion

This study shows four main results. First, IgG1 glycosylation differed between CSF and serum, even in the control group without intrathecal IgG synthesis. Second, in MS patients vs. controls, the IgG1 glycosylation pattern was altered in CSF, but not in serum. Third, alterations of glycosylation occurred especially shortly after a relapse. Fourth, glycosylation patterns in CSF from MS cases correlated with the degree of intrathecal IgG1 synthesis and CSF cell counts.

### Implications of CSF IgG1 glycosylation patterns for their effector functions

IgG glycosylation regulates Fc effector functions. A pro-inflammatory pattern consists of elevated bisecting GlcNAc but reduced galactosylation [45], as we observed for IgG from CSF of MS patients compared to controls. The functional relevance of IgG Fc glycosylation patterns has been shown in animal models of systemic autoimmune diseases [10–14] and is consistent with observations in serum from humans with autoimmune diseases. The decreased IgG galactosylation in CSF in MS we describe here parallels a similar observation of reduced galactosylation of anti-citrullinated protein antibodies in synovial fluid in rheumatoid arthritis [46], as well as reduced serum IgG galactosylation in rheumatoid diseases [47]. There is little data on autoimmune diseases and IgG containing bisecting GlcNAc, but they were also elevated in the serum of LEMS patients [24]. In addition, reduced sialylation is thought to be a pro-inflammatory feature [8], which did not reach statistical significance in our cohort, possibly because of the overall low degree of sialylation. Taken together, these alterations we describe here in CSF IgG1 from MS patients (reduced galactosylation, increased bisecting GlcNAc) suggest that the CSF IgG1 in MS patients has enhanced IgG effector functions, resulting in a higher pro-inflammatory activity than that of controls.

For the decrease of afucosylation, as observed in CSF IgG1 from MS patients, the interpretation is more complex; afucosylation has initially been regarded as pro-inflammatory by enhancing ADCC via FcγRIIIa [45]. A recent report confirmed this ADCC-enhancing effect of afucosylated IgG, but also reported an *opposite* effect, namely reduction of complement activation by afucosylation in the case of a therapeutic CD20 depleting antibody [22]. Thus, the decrease of afucosylation in the CSF of MS patients, compared to controls, may result in enhanced complement activation, but less ADCC. In fact, lesional complement activation was reported in the majority of RR-MS patients with early active lesions [6], and in an EAE model, autoantibody-mediated demyelination depended on complement activation but not on activatory Fc-receptors [48]. Nevertheless, Fc gamma receptors show elevated expression in MS lesions on microglia, albeit their exact role in MS is incompletely understood [49]. Therefore, the net outcome of the decrease of afucosylation in the MS group cannot be judged definitely, and we cannot exclude that it might also play a regulatory role for FcγRIIIa activation. In any case, the decrease of afucosylation in MS paralleled the findings in anti-citrullinated protein antibodies in rheumatoid arthritis [28].

Of note, MS-related changes in CSF IgG glycosylation where not only evident on the *inter*-group-level, but were linked to signs of inflammation also *within* the MS group, especially that the alteration in afucosylation (and as a trend also in galactosylation and bisecting GlcNAc) was more pronounced in those patients with stronger signs of intrathecal inflammation (higher CSF cell count and intrathecal IgG production).

When all four glycosylation features were combined by principal component analysis, separation of the groups was of similar discriminatory power as established markers such as CSF cell count or the intrathecal IgG fraction. However, there is no evidence that this inflammatory pattern of glycosylation is MS specific, but might rather be associated with the degree of inflammation. Using the glycopatterns and type of analysis we describe here, future studies can now apply these methods to address a number of obvious issues, such as the predictive value in addition to oligoclonal bands, comparison of autoimmune and infectious CNS diseases, alterations during aging, and effects of immunotherapy.

### Potential cause of altered IgG glycosylation

We conclude from our data that it is the intrathecal production of IgG that results in an altered glycosylation pattern for the following reasons. First, MS-related glycosylation abnormalities were present in CSF, but not in blood. Second, the degree of these abnormalities (esp. afucosylation) in the CSF from MS patients correlated with the fraction of IgG that was actually produced intrathecally (IF_IgG_). Third, the strong correlation of CSF and serum IgG glycosylation in control donors was much weaker in the MS group, where intrathecally produced IgG occurs in addition to circulation-derived IgG. Fourth, the group differences between controls and MS patients were greater for IgG1 than for IgG2, and intrathecally produced IgG is mainly IgG1 [50, 51].

Previous cell culture experiments have provided evidence that the cytokine milieu and pH determine the glycopattern of secreted IgG [52, 53]. Thus, the inflammatory milieu in the MS CNS is a likely cause for the pro-inflammatory glycofeatures of locally produced IgG that we observed in the CSF of patients with MS. This could constitute a positive feedback loop for CNS inflammation, reminiscent of a loop between dys-glycosylated myelin and inflammation [54].

Of note, IgG1 afucosylation and the presence of bisecting GlcNAc peaked 2–3 months after a clinical relapse. Even though the half-life of IgG1 is uncertain in the CSF (serum; 21 days), the kinetics were compatible with an inflammatory glycosylation pattern building up at the time of a relapse. It is possible that incorporating data on subclinical MRI activity and lesion localization would result in even better correlation of glycosylation patterns and disease features, but frequent MRI data were not available.

IgG glycosylation features were largely independent of each other within the same group and compartment, except in the case of sialylation and galactosylation, which highly correlated with each other. This seems plausible, given the subsequent addition of terminal sialic acid on top of galactose (Fig. 1). However, since sialylation is much lower than galactosylation, there would be enough space for independent degrees of sialylation and galactosylation. Therefore, the strong correlation of sialylation and galactosylation suggests that these two glycosyltransferases might be regulated in parallel, whereas regulation of the other MS-related glycosylation features may be mechanistically different.

### Even in the normal CSF, IgG glycosylation is distinct from serum

An unexpected finding in this study was that IgG glycosylation in the CSF is distinct from serum even in the absence of inflammation and intrathecal IgG production. Although these differences were small, they were significant also after adjustment for multiple testing. Potential explanations include that (1) IgG transport into or (2) half-life within the CSF compartment depends on glycosylation, or that (3) IgG glycosylation is modified in the CSF or serum.

Galactosylation was reduced in control CSF and could thus favor IgG effector functions also in healthy subjects, but it is likely that a further reduction of galactosylation, as observed in MS (Fig. 4), as well as the presence of complement and cells that mediate ADCC, which are present in MS lesions, are necessary to actually unleash IgG effector functions. Of note, the pattern of differences between control CSF and serum, and those between MS and control CSF, were not identical; in particular, afucosylation was increased in control CSF vs. serum but decreased in MS vs. control CSF.

### Limitations of this study

Since clinical samples were collected at different sites, we cannot completely rule out any site bias. However, in order to minimize any pre-analytical issues, samples were collected following the same protocol, centrifuged and frozen immediately, shipped on dry ice, and analyzed by mass spectrometry altogether as detailed in the Methods section. Analyzing the CSF/serum ratios of the MS vs. the control group separately for site 1 and site 2, we observed similar changes also within the samples of site 1 or 2 for the glycofeatures with significant regulation, arguing against a major site bias.

## Conclusions

The CNS compartment as well as the inflammatory milieu in MS affect IgG1 Fc glycosylation. In MS, the altered CSF IgG1 glycosylation pattern has pro-inflammatory features and is linked to intrathecal IgG synthesis (see also Fig. 1 and Table 2 for summarizing depiction; Fig. 6). We suggest that the inflammatory intrathecal milieu in MS might cause the described pro-inflammatory IgG glycosylation pattern, which in turn might further support pro-inflammatory IgG effector mechanisms, possibly constituting a vicious circle that helps to perpetuate the inflammatory process in MS.

## Additional files

Additional file 1: Figure S1. Representative mass spectrometric IgG1 and IgG2 Fc glycosylation data from an MS patient. Tryptic Fc glycopeptides of IgG1 and IgG2 isolated from (A) serum and (B) cerebrospinal fluid (CSF) from a MS patient were analyzed by MALDI-FTICR-MS. IgG1 (continued arrow) and IgG2 (striated arrow) glycopeptide signals with identical glycan portions were registered as peak pairs due to a 32-Da mass difference of the peptide moieties. The inset shows the signals obtained for two sialylated glycopeptide species. *pep* peptide moiety. Symbols and colors are drawn according to the *Consortium for Functional Glycomics* [55]. (PDF 236 kb) Additional file 2: Figure S2. CSF vs. serum IgG2 glycosylation. Afucosylation could only be assessed for IgG1, but not for IgG2, as several fucosylated IgG2 glycoforms could not be determined due to overlay with IgG4 glycan structures. Individual data points are horizontally jittered to avoid obscuring them from each other. Lines indicate corresponding CSF/serum pairs but do not necessarily end directly at the horizontally jittered data points to preserve angles of the connecting lines. Significance was determined using Wilcoxon-signed rank test for paired samples, followed by Bonferroni correction for multiple testing (*p*_ad_j). Factors above diagrams indicate fold-changes (medians of paired CSF/serum ratios). (PDF 326 kb) Additional file 3: Figure S3. IgG2 glycosylation in CSF and serum from MS patients vs. controls. CSF IgG2 glycosylation (normalized to serum IgG2 glycosylation) is displayed. Significance was determined using Mann-Whitney *U* test, followed by Bonferroni correction for multiple testing (*p*_ad_j). Factors above diagrams indicate fold-changes. IgG2 afucosylation could not be assessed because of overlay with IgG4 glycan structures. (PDF 169 kb) Additional file 4: Figure S4. Terminal IgG1 sialylation and galactosylation correlate with each other (A), whereas other combinations of glycosylation features within the same group and compartment do not (B). *ϱ* and p values (Spearman’s method, adjusted for multiple testing) are given for each diagram. Trendlines represent LOWESS lines (Cleveland 1979) and indicate the strength of association by their opacity and thickness. (PDF 254 kb) Additional file 5: Table S1. IgG2 vs. IgG1 glycosylation. Displayed are ratios (IgG2/IgG1) for each glycofeature within the same group and compartment. *p*_adj_ denotes the *p* value after Bonferroni correction. IgG2 afucosylation could not be assessed because of overlay with IgG4 glycan structures. (DOC 32 kb)

## Abbreviations

CSF: cerebrospinal fluid
GlcNAc: N-acetylglucosamine
IgG: immunoglobulin G
MS: multiple sclerosis
Fc: fragment crystallizable
FcγR: Fc gamma receptor
CH_2_: constant heavy domain 2
