# Supplementary material for: Pro-inflammatory pattern of IgG1 Fc glycosylation in multiple sclerosis cerebrospinal fluid
Source: J Neuroinflammation. 2015 Dec 18;12:235. doi: 10.1186/s12974-015-0450-1 (PMC4683913; doi:10.1186/s12974-015-0450-1)
Supplement: Additional file 1: Figure S1. — Representative mass spectrometric IgG1 and IgG2 Fc glycosylation data from an MS patient. Tryptic Fc glycopeptides of IgG1 and IgG2 isolated from (A) serum and (B) cerebrospinal fluid (CSF) from a MS patient were analyzed by MALDI-FTICR-MS. IgG1 (continued arrow) and IgG2 (striated arrow) glycopeptide signals with identical glycan portions were registered as peak pairs due to a 32-Da mass difference of the peptide moieties. The inset shows the signals obtained for two sialylated glycopeptide species. pep peptide moiety. Symbols and colors are drawn according to the Consortium for Functional Glycomics [55]. (PDF 236 kb) [file 12974_2015_450_MOESM1_ESM.pdf]

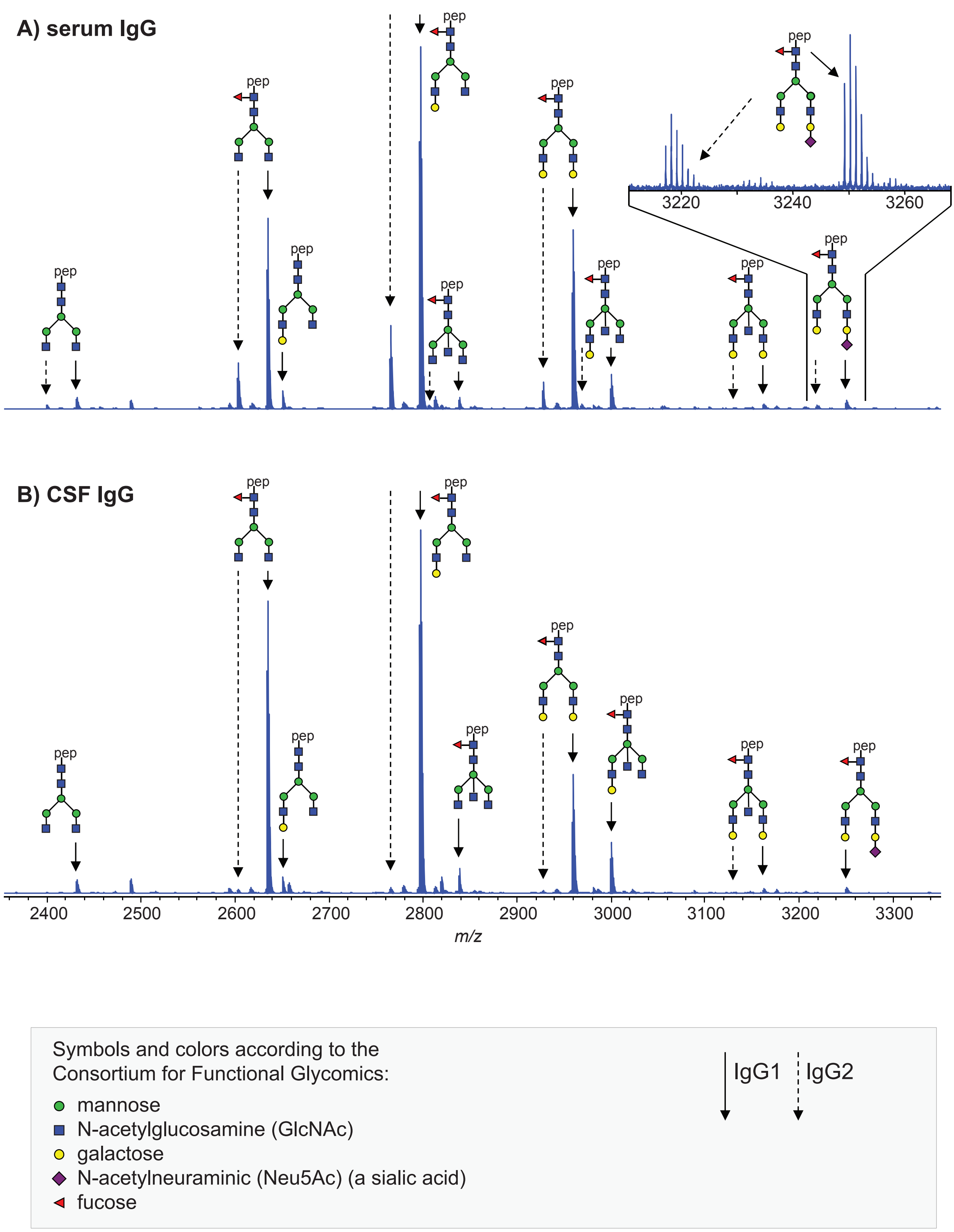
A) serum IgG
B) CSF IgG
pep
3220
3240
3260
2400
2500
2600
2700
2800
2900
3000
3100
3200
3300
m/z
Symbols and colors according to the Consortium for Functional Glycomics:
mannose
N-acetylglucosamine (GlcNAc)
galactose
N-acetylneuraminic (Neu5Ac) (a sialic acid)
fucose
IgG1
IgG2
