# Supplementary figures and images for: Pro-inflammatory pattern of IgG1 Fc glycosylation in multiple sclerosis cerebrospinal fluid
Source: J Neuroinflammation. 2015 Dec 18;12:235. doi: 10.1186/s12974-015-0450-1 (PMC4683913; doi:10.1186/s12974-015-0450-1)

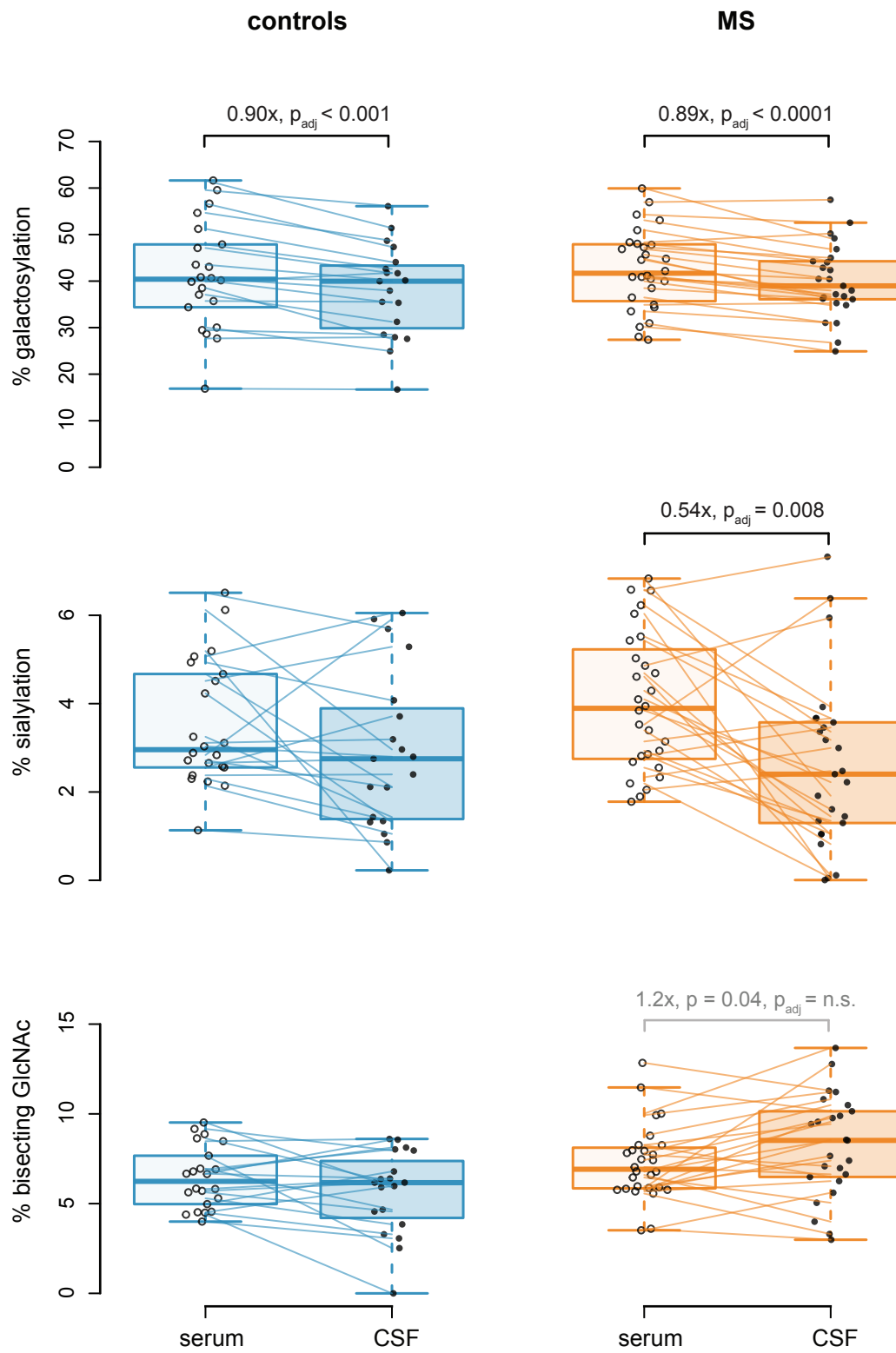

Supplement: Additional file 2: Figure S2. — CSF vs. serum IgG2 glycosylation. Afucosylation could only be assessed for IgG1, but not for IgG2, as several fucosylated IgG2 glycoforms could not be determined due to overlay with IgG4 glycan structures. Individual data points are horizontally jittered to avoid obscuring them from each other. Lines indicate corresponding CSF/serum pairs but do not necessarily end directly at the horizontally jittered data points to preserve angles of the connecting lines. Significance was determined using Wilcoxon-signed rank test for paired samples, followed by Bonferroni correction for multiple testing (p adj). Factors above diagrams indicate fold-changes (medians of paired CSF/serum ratios). (PDF 326 kb) [file 12974_2015_450_MOESM2_ESM.pdf]

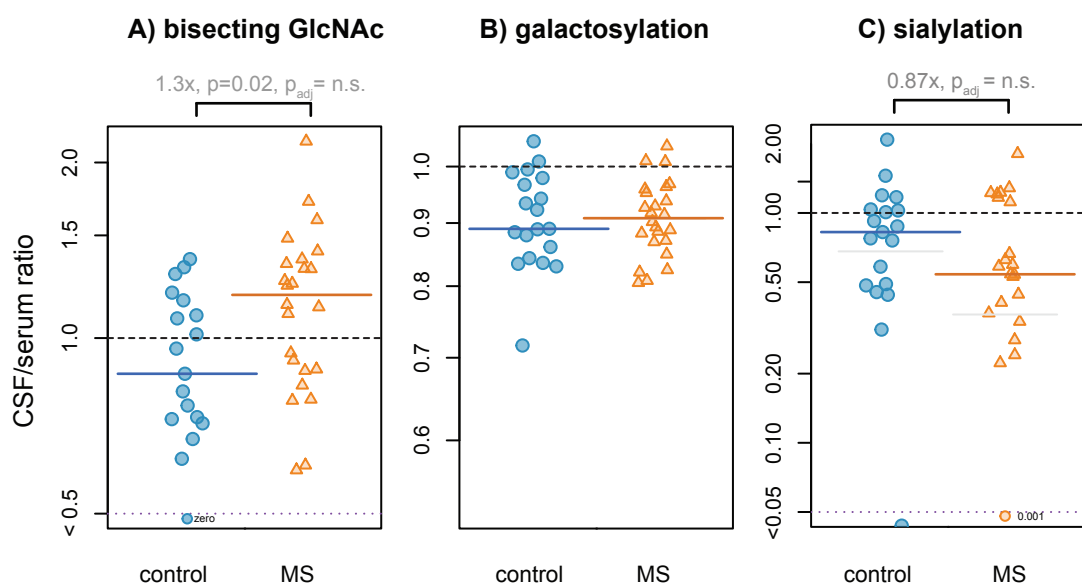

Supplement: Additional file 3: Figure S3. — IgG2 glycosylation in CSF and serum from MS patients vs. controls. CSF IgG2 glycosylation (normalized to serum IgG2 glycosylation) is displayed. Significance was determined using Mann-Whitney U test, followed by Bonferroni correction for multiple testing (p adj). Factors above diagrams indicate fold-changes. IgG2 afucosylation could not be assessed because of overlay with IgG4 glycan structures. (PDF 169 kb) [file 12974_2015_450_MOESM3_ESM.pdf]
