# Supplementary material for: Pro-inflammatory pattern of IgG1 Fc glycosylation in multiple sclerosis cerebrospinal fluid
Source: J Neuroinflammation. 2015 Dec 18;12:235. doi: 10.1186/s12974-015-0450-1 (PMC4683913; doi:10.1186/s12974-015-0450-1)

Suppl. Fig. 4: Terminal IgG1 sialylation and galactosylation correlate with each other, whereas other combinations of glycosylation features within the same group and compartment do not.

A) Significant correlation of sialylation with galactosylation

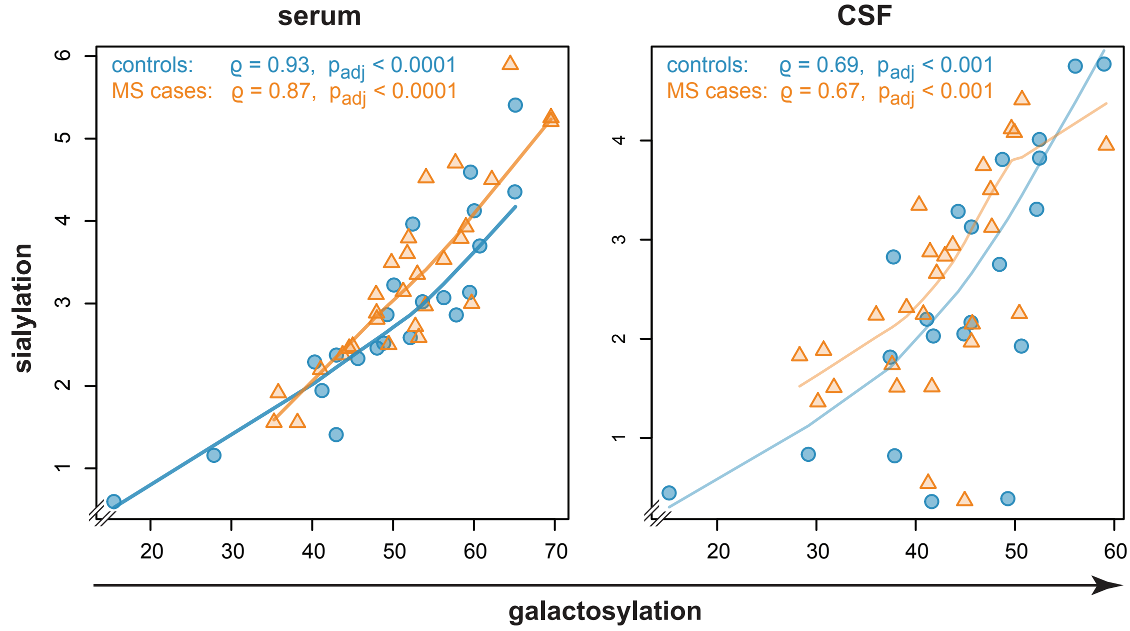

B) No correlation of other glycofeatures

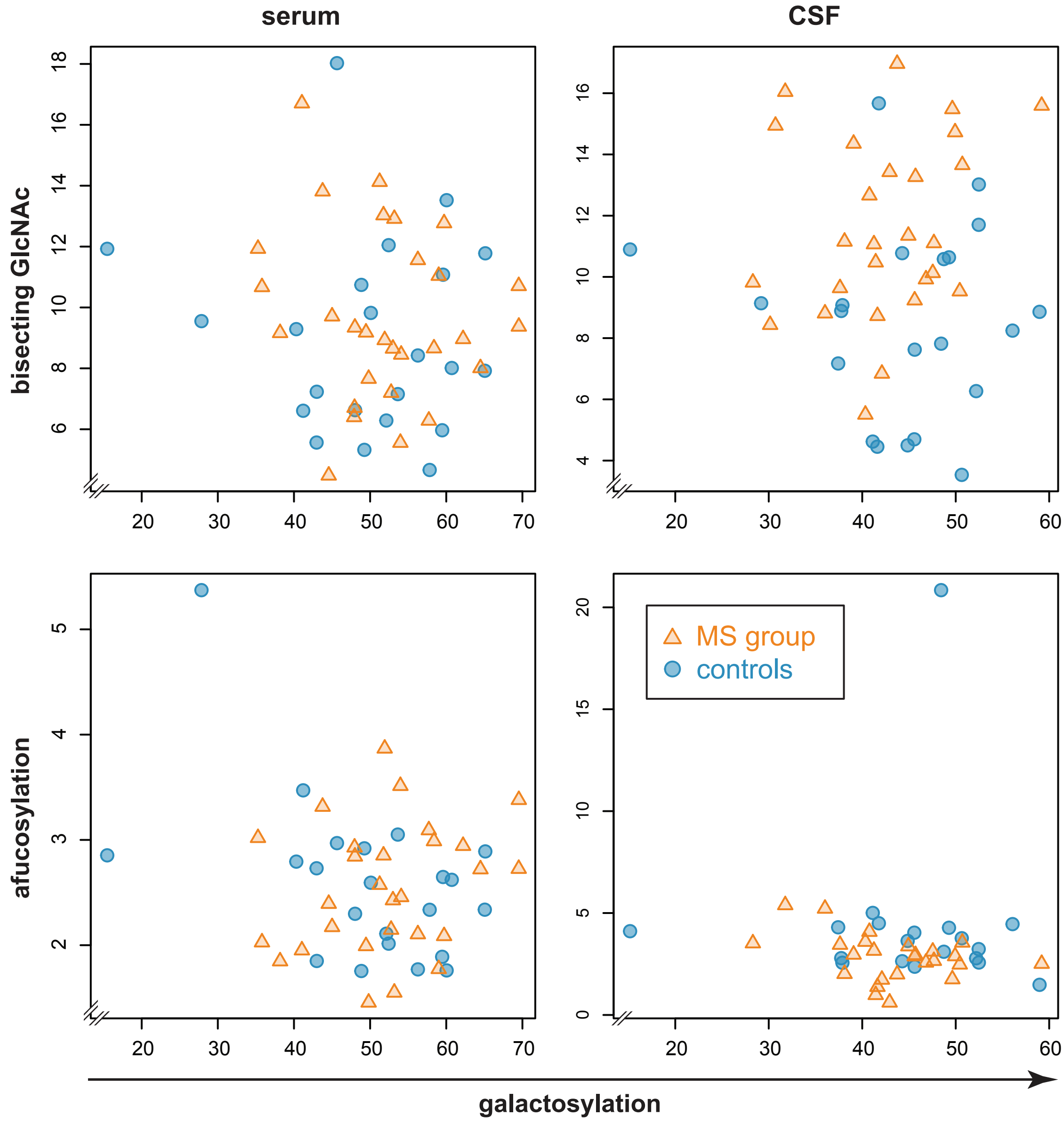

Supplement: Additional file 4: Figure S4. — Terminal IgG1 sialylation and galactosylation correlate with each other (A), whereas other combinations of glycosylation features within the same group and compartment do not (B). ϱ and p values (Spearman’s method, adjusted for multiple testing) are given for each diagram. Trendlines represent LOWESS lines (Cleveland 1979) and indicate the strength of association by their opacity and thickness. (PDF 254 kb) [file 12974_2015_450_MOESM4_ESM.pdf]
