# Supplementary material for: Pro-inflammatory pattern of IgG1 Fc glycosylation in multiple sclerosis cerebrospinal fluid
Source: J Neuroinflammation. 2015 Dec 18;12:235. doi: 10.1186/s12974-015-0450-1 (PMC4683913; doi:10.1186/s12974-015-0450-1)
Supplement: Additional file 5: Table S1. — IgG2 vs. IgG1 glycosylation. Displayed are ratios (IgG2/IgG1) for each glycofeature within the same group and compartment. p adj denotes the p value after Bonferroni correction. IgG2 afucosylation could not be assessed because of overlay with IgG4 glycan structures. (DOC 32 kb) [file 12974_2015_450_MOESM5_ESM.doc]

Supplementary Table

**Suppl. Table 1: IgG2 *vs.* IgG1 glycosylation.** Displayed are ratios (IgG2/‌IgG1) for each glycofeature, within the same group and compartment. p_adj_ denotes the p value after Bonferroni correction. IgG2 afucosylation could not be assessed because of overlay with IgG4 glycan structures.

|  | **MS group** | | **controls** | |
| --- | --- | --- | --- | --- |
|  | serum | CSF | serum | CSF |
| bisecting GlcNAc | ↓ 0.7  p_adj_ <0.0001 | ↓ 0.7   p_adj_ =0.0001 | ↓ 0.7  p_adj_ =0.0003 | ↓ 0.7  p_adj_ =0.002 |
| galactosylation | ↓ 0.8  p_adj_<0.0001 | (↓) 0.9   p=0.02, p_adj_  n.s. | ↓ 0.8   p_adj_<0.0001 | ↓ 0.8   p_adj_<0.0001 |
| sialylation | ↑ 1.2  p_adj_=0.0005 | 0.9   (n.s.) | ↑ 1.2   p_adj_=0.02 | 1.0   (n.s.) |
